# Knowledge, attitudes, and practices of caregivers on childhood immunization in Okaikoi sub-metro of Accra, Ghana

**DOI:** 10.3389/fpubh.2023.1230492

**Published:** 2023-09-15

**Authors:** Samuel E. Danso, Augustina Frimpong, Nana A. H. Seneadza, Michael F. Ofori

**Affiliations:** ^1^Department of Community Health, University of Ghana Medical and Dental School, College of Health Science, Accra, Ghana; ^2^GA East Hospital, Accra, Ghana; ^3^Department of Immunology, Noguchi Memorial Institute for Medical Research, College of Health Sciences, University of Ghana, Accra, Ghana

**Keywords:** immunization, child immunization, attitudes, Ghana, immunization coverage

## Abstract

**Background:**

Immunization remains one of the most cost-effective health interventions. However, there are still issues of vaccine hesitancy especially in caregivers who are required to protect their children from vaccine-preventable diseases. This thwarts the overall vaccine coverage in disease-endemic areas such as sub-Saharan Africa. Therefore, to determine the factors that promote vaccine hesitancy in caregivers, this study sought to assess the knowledge, attitude, and practices of caregivers on childhood immunization in Okaikoi, a sub-metro of Accra in Ghana.

**Methods:**

A cross-sectional study on childhood immunization was conducted to determine the knowledge, attitudes, and practices of caregivers. A total of 120 caregivers with infants aged 12 months to 23 months were interviewed with a structured questionnaire containing open-ended and closed-ended queries.

**Results:**

From the community, infants whose caregivers had adhered completely to immunization constituted 53.3% while the rest were partially immunized. The two main deterrents to complete immunization were time constraints (25.8%) and forgetfulness (17.5%). It was observed that vaccination uptake and maternal level of education, as well as vaccination adverse reaction, did not impact the completion of the EPI program by these caregivers. Unfortunately, it was noted that caregivers with higher education levels were unable to complete their vaccination schedules due to their busy work schedules. Nonetheless, the main deterrent to adhering to complete childhood immunization was poor maternal knowledge (58%).

**Conclusion:**

The study revealed that, the caregivers in the community had poor knowledge on vaccination and its benefits, and therefore, with no strict adherence to vaccination schedules. This promoted the incomplete immunization of children in the community by their caregivers. Also, since the main source of information with regard to immunization in the sub-metro was through the antenatal and postnatal child welfare clinics and the media, we recommend that the health workers collaborate with media personnel to ensure that standardized information is disseminated.

## Background

Immunization is a low-cost approach to averting debility and disease from vaccine-preventable diseases (VPD). Active immunization averts over 2 million deaths each year in developing countries (1). Importantly, childhood immunization has been reported to provide herd immunity by preventing infectious diseases in the adult population (2). However, vaccine uptake has been decreasing gradually in these areas due to parent’s failure to vaccinate their children (3, 4). Nevertheless, VPD remains a major cost of morbidity and mortality in children under 5 years of age in developing areas including Africa (5, 6). For instance, in 2014, it was estimated that the WHO African region accounted for a higher proportion of 33% of the worldwide cases of pertussis (7). Therefore, increasing the coverage of childhood vaccinations in Africa is a requirement to achieve the Global Vaccine Action Plan targets (8, 9). Most sub-Saharan African countries including Ghana have very low childhood immunization coverage despite improvement over the past years (10). For Ghana to achieve the Sustainable Development Goal (SDG) 3, of ending preventable deaths of children under 5 years of age by 2030, routine vaccination structures should be considered and constructed since infants denied routine vaccination have been found to be 6 times more prone to acquiring pertussis than infants who were immunized. Additionally, they were even more likely to contract measles, a disease with debilitating infections (11).

It has been reported that inadequate knowledge of immunization and the perception of caregivers are among the hindrances to vaccination uptake (12, 13). A major contributor to achieving vaccination coverage and uptake in Ghana is equipping mothers with the prerequisite knowledge about the benefits of immunization. This might impact their attitude and encourage their participation in vaccination services. Essentially, this also depends primarily on the public’s knowledge of vaccine-preventable diseases and the availability and accessibility of these immunization services. Additionally, knowing the sources of these caregiver’s information on vaccination will help the health system in the country to prioritize the areas that need further improvement. Unfortunately, this has been a stumbling block in sub-Saharan Africa, where policymakers ignore awareness, knowledge, and conditions of non-immunized and partially immunized populaces (14).

According to the Ghana Demographic Healthy Survey (GDHS) 2014, despite efforts to increase vaccination coverage by 90% nationwide in Ghana, there was a marginal drop from 79% in 2012 to 77% in 2014. Additionally, this society is also naturally inclined to traditional herbal treatment due to gross superstition concerning the orthodox medical practice which in recent times concentrates on both curative and preventive healthcare delivery systems (15, 16).

This study was aimed at determining the basic knowledge and attitude of mothers about immunization and how these affect the full immunization status of their children. This study, therefore, recruited parents or guardians of infants aged 12 months to 23 months to determine the knowledge, attitude, and practices of mothers or guardians of childhood immunization in Ghana. It is believed that such information will boost the efforts of health workers in Ghana to improve childhood immunization in Ghana.

## Methods

### Ethical consideration

The study protocol was reviewed and approved by the Ethical and Protocol Review Committee of the University of Ghana Medical School, Accra, Ghana (UGMS-CHDRC/077/2016). Permission was first sought from opinion leaders of the Okaikoi Sub-Metro of Accra with the aid of the Public Health Nurses who work in the community. Then, written informed consent was obtained from the study participants (caregivers) by providing the purpose of the study and the assurance of confidentiality. Involvement in the study was voluntary and participants were given the choice to withdraw from the study at any time. Questionnaires were administered to recruited participants and the completed questionnaires were kept under strict confidentiality.

### Study design

This was a cross-sectional community-based study that used quantitative techniques to obtain information from respondents. The interview was conducted in English language and was translated into the local language (Twi or Ga) for caregivers who could not use the English language.

### Study area

The study was carried out in the Okaikoi South sub-metro, one of the 13 constituencies of the Greater Accra Region in Ghana. The sub-metro is situated in the western part of the city of Accra, covers an area of 24 km^2^, and has a population of 340,380, with a population density of 14,183 persons/km^2^. The Kaneshie Polyclinic located in the Okaikoi South sub-metro was established in 1964 and was the first to be built in Greater Accra. There are 18 private hospitals and clinics, two (2) quasi-government clinics (Cocoa Clinic and Police Depot Clinic), two (2) Christian Health Association clinics, and three (3) private maternity homes. The Okaikoi sub-metro is an old community and other Ghanaian tribes are marginal. The inhabitants are civil and public servants, entrepreneurs, traders, and a few farmers. The inhabitants accept both conventional and herbal medicine.

### Sampling strategy

The Okaikoi sub-metro has five zones. The names of the zones were written on small pieces of paper and the Abeka zone was randomly selected through balloting. Two communities under the Abeka zone, Nii Boi town and Abeka, were also selected by balloting. Each selected community was divided into two blocks and a block was also selected using balloting. Then, within the selected block, the number of houses to be included in the study was selected using the formula below: K = N/nwhere *N* is the number of houses within the selected block [360].

*n* is the sample size [120].

A house number 1 and *K*-value thus house numbered 2 was selected for the interview.

Then, every *K*th (thus every second house) house was interviewed for the study. If the subsequent second house had no caregiver with a child aged 12 months to 23 months, then another block was selected by balloting. Together, a sample size of 120 mothers or guardians (caregivers) with infants aged 12 months to 23 months were selected. The sample size was chosen because of the convenience of time and money.

### Study population

Caregivers of children aged 12–23 months, who were residents in the Okaikoi South sub-metro (Abeka zone) in the Greater Accra Region of Ghana, were recruited for the study. This age group represents the youngest cohort of infants qualified for the Expanded Program on Immunization (EPI) in Ghana.

### Data collection instruments and methods

The study questionnaires were in three parts. The first part was on the participant’s demographics, the second part was on the knowledge of childhood immunization, and the third was on the caregiver’s attitude and practice toward vaccination. The knowledge and attitude of study participants were examined using both closed and open-ended queries contained in structured or dichotomous and multiple choices. Knowledge was assessed using a 3-point scale. Higher scores indicated good knowledge, median scores indicated moderate knowledge, and lower/no scores indicated less or no knowledge (17, 18). The study questionnaires were prepared in English and later translated verbally into the local languages where necessary. Questionnaires were administered and interviews were conducted face-to-face with study participants.

### Statistical analysis

The data were analyzed using the Statistical Package for the Social Sciences [SPSS] and Microsoft Excel. The results of the study were represented using cross tabs, tables, bar graphs, and pie charts.

## Results

### Characteristics of study participants

A total of 120 caregivers were enrolled in this study with each parent having one child at the age for immunization. Fifty of the infants representing 41.7% were males and 58.3% were females. These caregivers were interviewed to assess factors associated with the completion of childhood immunization. The group of children enrolled in this mother–child pair was less than 24 months old. Most of these caregivers were between 25 and 29 years of age, representing 49.2%, whereas the proportion of respondents aged below 20 years constituted 2.5%. Moreover, most of these women were married with less than 6% living as single parents (Table 1). Most caregivers were Christians with the second most common religion being Islam. All caregivers had postnatal books that were used for records during childhood immunizations at health facilities or immunization centers.

**Table 1:** Demographic characteristics of caregivers and their children enrolled in the study.

Age of child (months)	*n* (%)
12–14	25
15–17	23.3
18–20	35
21–23	16.7
**Sex of child**	
Male	41.7
Female	58.3
**Age of mother (years)**	
<20	2.5
20–24	15.8
25–29	49.2
30–34	22.5
35–39	6.7
>39	3.3
**Marital status**	**%**
Married	75
Co-habiting	19.2
Never married	5
Divorce	0.8
**Religious affiliation**	**%**
Christianity	77.5
Islamic	19.2
Traditionalist	1.7
Pagan	1.7

**Figure 1:**
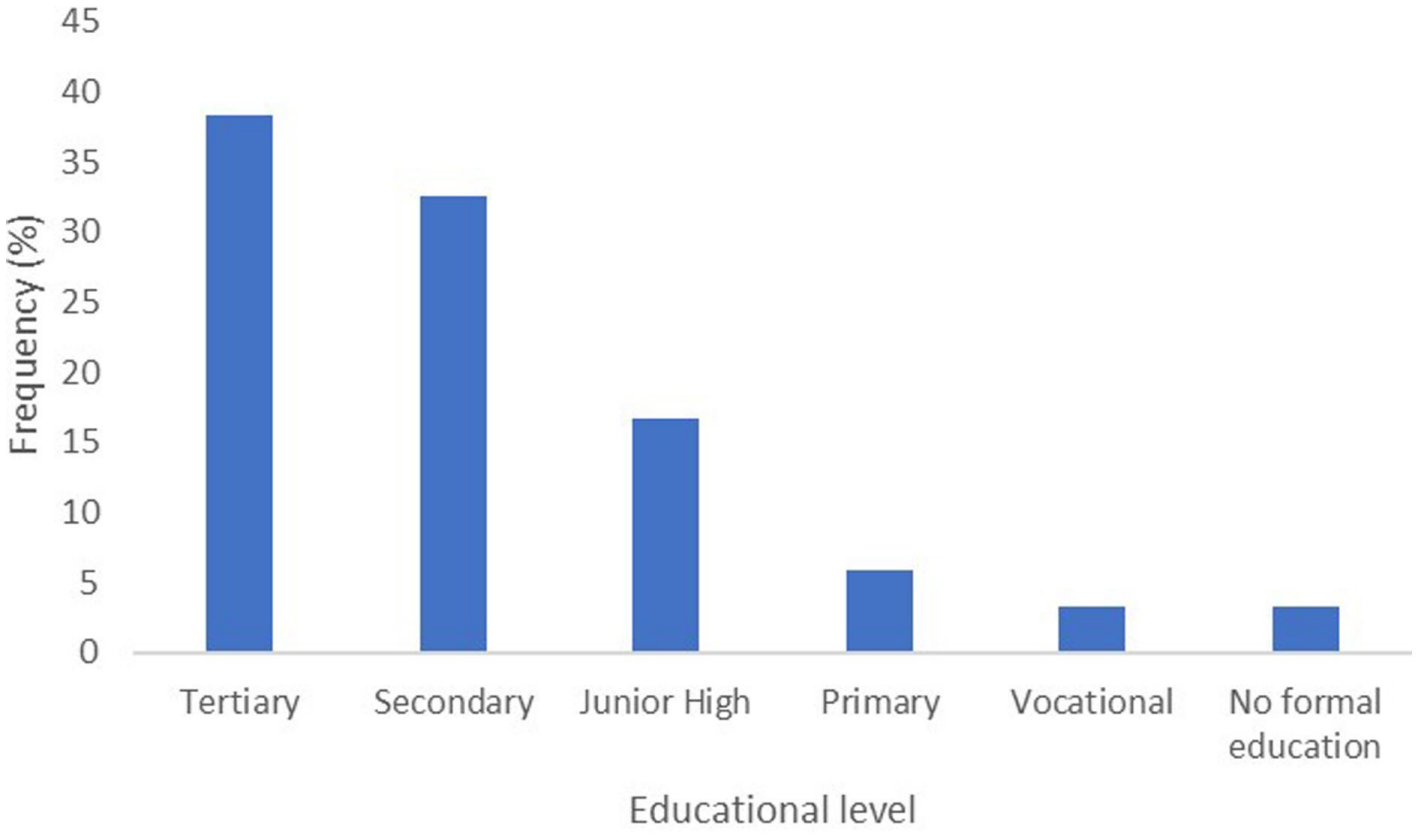
Distribution of the highest education of caregivers. A bar chart of the educational level of respondents. The graph shows that 3.3% of the respondents had no formal education, whereas the percentage of respondents with tertiary education was 38.3%.

We also assessed the educational status of the participants as well as their current occupation. We found that more than 80% of the women had some form of formal education with approximately 38% completing tertiary education (Figure 1). In addition, 25.9% did not go beyond the basic (Primary and Junior High school) level and 58.4% of respondents did not go beyond the Senior Secondary school level. The remaining 3.3% had no form of formal education. Interestingly, only 30% of these women were public servants with approximately 32.5% being traders and the remaining being in other vocational trades or jobs (Table 2).

**Table 2:** Distribution of caregivers’ occupation.

Occupation	%
Trader	32.5
Public servant	30
Vocational jobs	21.7
Housewife	10
Student	5.8

### Assessment of the knowledge of vaccination in caregivers

The levels of the caregivers’ knowledge of vaccination are shown in Table 3. During the interviews, all the respondents stated that immunization was useful and showed a moderate level of knowledge of vaccination. Of the 120 respondents, 113 representing 94.2% of the respondents believed that childhood immunizations protected their children from diseases. The rest gave the following reasons for childhood immunization: making children brilliant (3.3%), curing diseases (1.7%), and less than 1% stated that immunization makes children stronger.

**Table 3:** Mother’s perceived knowledge of childhood immunization.

Reasons for vaccination			*n* (%)
Disease prevention			94.2
Curing diseases			1.7
Makes children brilliant			3.3
Makes children stronger			0.8
**Source of information about vaccination**			*n* **(%)**
Health center (antenatal and child welfare clinic)			80.8
School			10.8
Family			1.7
Friends			3.3
**Diseases prevented by vaccination**			
**Diseases**	**Yes (%)**	**No (%)**	**Do not know (%)**
Measles	80.8	–	19.2
Whooping cough	38.3	9.2	52.5
Tuberculosis	52.5	11.7	35.8
Diphtheria	36.7	5.8	57.5
Poliomyelitis	84.2	–	15.8
Tetanus	80.8	5.8	13.4
Yellow fever	60.8	1.7	37.5
Hepatitis B	55	5	40
Malaria	31.7	43.3	25
Cholera	25.8	47.5	26.7
HIV	18.3	59.2	22.5

A majority of the caregivers knew that vaccination could thwart Poliomyelitis, Measles, Tetanus, and Yellow fever. However, approximately half of these respondents did not know that diphtheria (57.5%), whooping cough (52.5%), and hepatitis B (40%) were vaccine-preventable diseases. Interestingly, some respondents thought malaria (31.7%) and HIV/AIDS (18.3%) were vaccine-preventable. The majority of caregivers (80%) stated that their knowledge of vaccination came from the health center where they visited for vaccinations (94%; Table 3).

**Figure 2:**
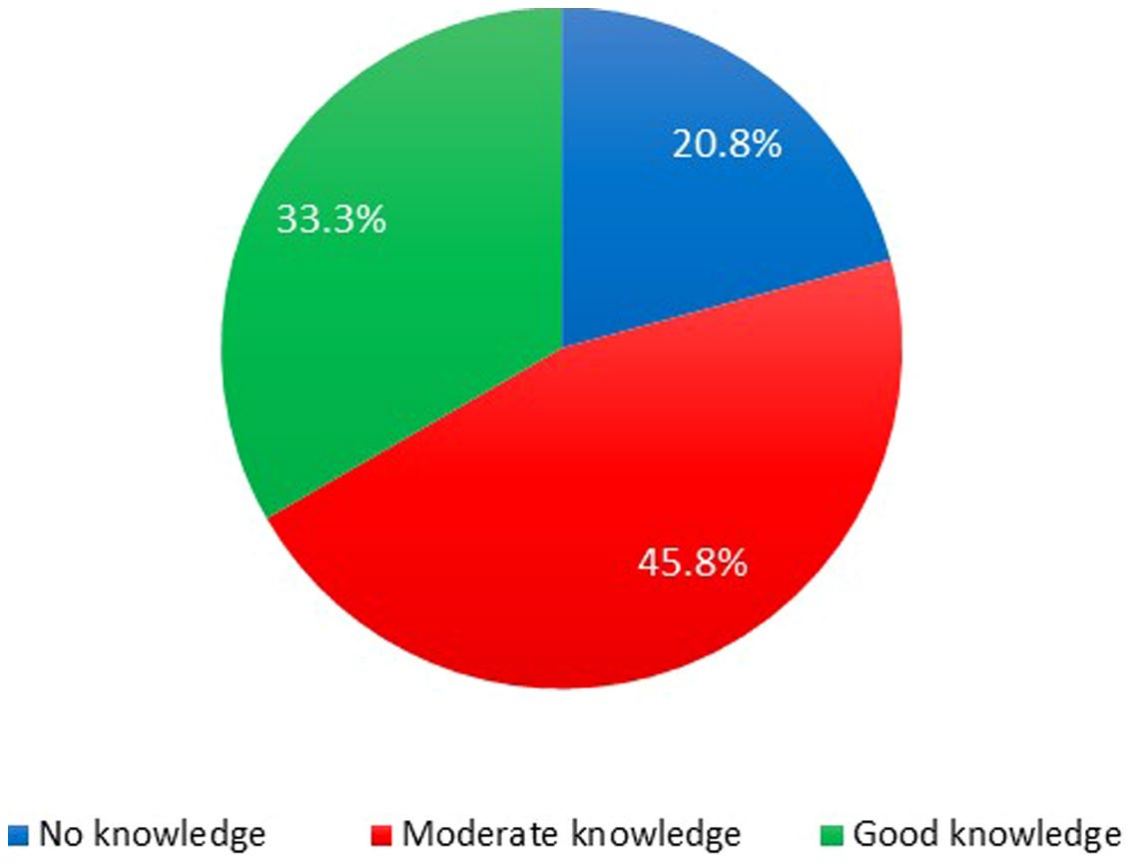
Percentage of respondents with correct knowledge regarding child’s age for immunizations. Approximately 76.7% of caregivers stated that they knew the ages at which their wards are to be immunized and the rest did not but upon assessing, only 33.3% had good knowledge of the ages for immunization, while 45.8% had moderate knowledge and 20.8% had no knowledge.

**Figure 3:**
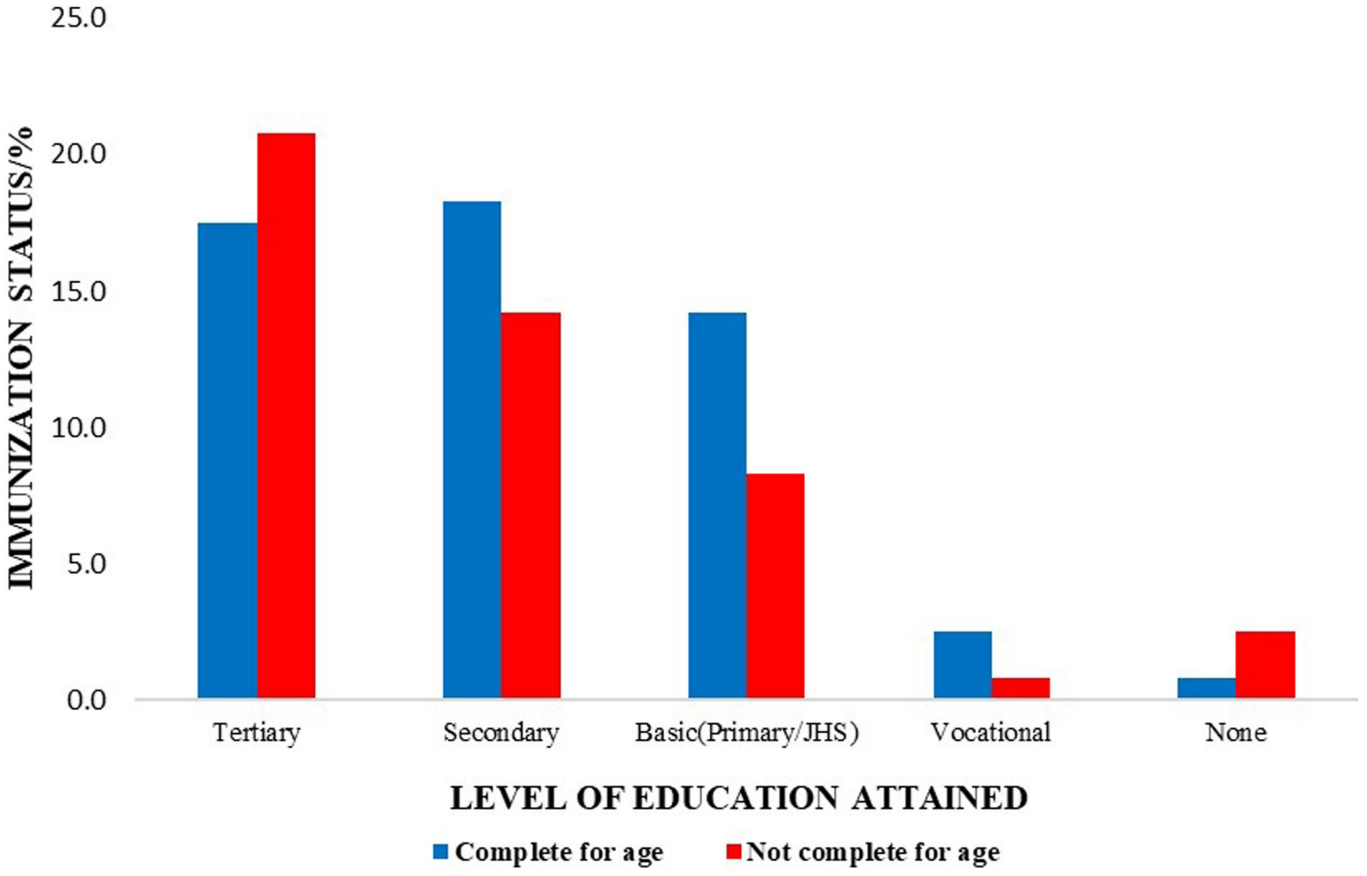
Major factors serving as a hindrance to vaccine uptake. Poor maternal knowledge and time constraints were the major hindrances to child immunization in the Okaikoi sub-metro.

With regard to the ages required for vaccination, only approximately 33% of caregivers knew when the children were to be sent for immunizations (Figure 2). Over 67% of the caregivers knew about vaccine adverse reactions with most of their information source being from healthcare workers (49.2%; Table 4). Importantly, we found that poor maternal knowledge, time constraints, and illiteracy were the most contributing factors affecting vaccine hesitancy (Figure 3).

**Table 4:** Sources of information on vaccination adverse reactions.

Information source	*n* (%)
Health workers	49.2
Personal experience	12.5
Family/friends	2.5
Media	3.3
N/A	32.5

### Assessment of caregivers’ attitude toward vaccination

**Figure 4:**
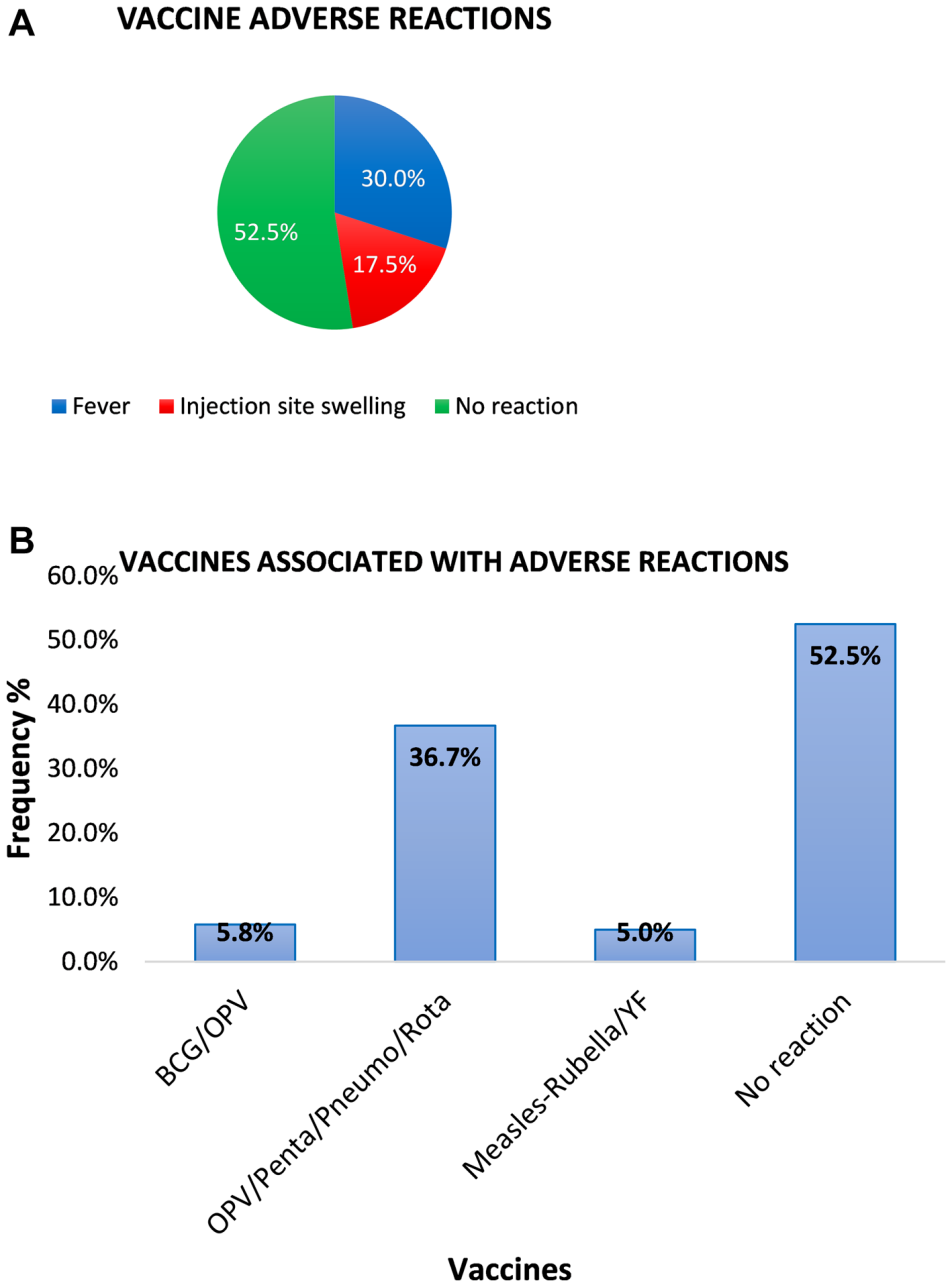
Types of common adverse reactions developed after immunization in infants of respondents. **(A)** The pie chart shows the percentage of infants and the common adverse reactions developed after vaccination. In total, 57 out of the 120 caregivers representing 47.5% reported that their wards developed vaccination adverse reactions, 40% did not report any vaccination reaction, and 12.5% were indifferent. **(B)** The majority of the respondents (36.7%) stated that the 6th-week, 10th-week, and 14th-week vaccines, oral polio 1, pentavalent, and Pneumovax, respectively, are associated with adverse reactions.

When asked what would affect their attitude toward vaccination, most of these caregivers cited vaccine adverse reactions (Figure 4A). Additionally, the majority of the respondents (36.7%) stated that the 6th-week, 10th-week, and 14th-week vaccines, oral polio 1, pentavalent (diphtheria, pertussis, tetanus, hepatitis B, and *Haemophilus influenzae* B), and Pneumovax, respectively, were associated with adverse reactions (Figure 4B). However, despite listing vaccine adverse reactions, it was observed that approximately 53.3% of the caregivers had completed the vaccinations required in the EPI program compared to 46.7% who failed to complete the vaccination requirement (partial vaccination). The main reasons for partial vaccination given were time constraints (55.4%), forgetfulness (37.5%), and that it was expensive (7.1%).

**Figure 5:**
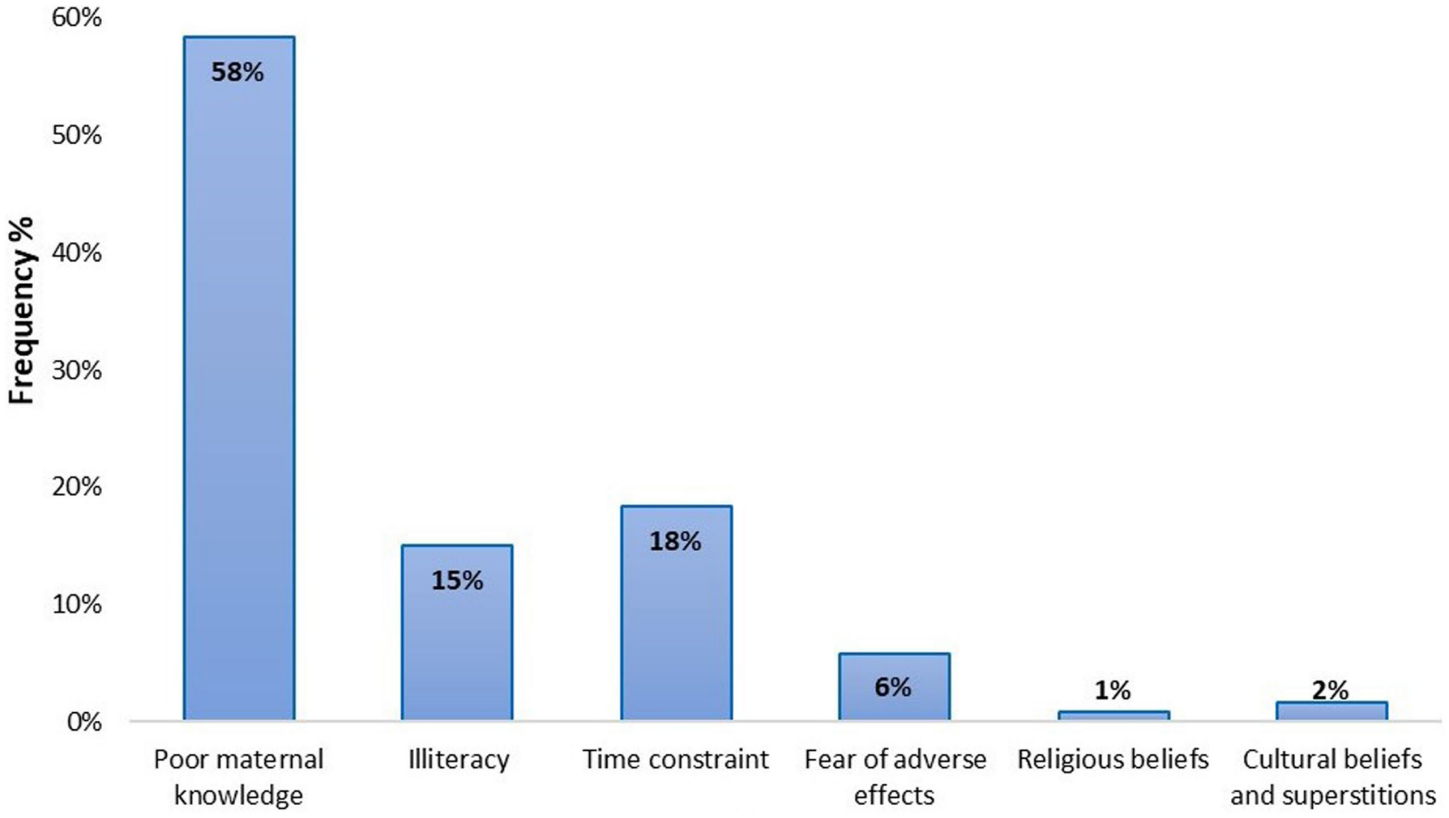
Comparison between mother’s educational level and children’s immunization status.

Furthermore, assessing the caregiver’s educational status in completing immunization schedules for their infants, the majority of the caregivers with secondary and basic education completed vaccination schedules compared to women with tertiary education (Figure 5).

## Discussion and conclusion

In this study, a total of 120 caregivers with children less than 23 months in a suburb in Accra, Ghana, were enrolled to assess their knowledge, attitude, and practices on immunization to determine the efficacy of the National Expanded Program of Immunization in Ghana. Generally, the results show that caregivers have a poor knowledge of vaccination which may likely contribute to vaccine hesitancy. Interestingly, we found out that highly educated caregivers were more likely to miss the vaccination schedules compared to those with less formal education. This is in contrast to other studies that have associated high vaccination rates among well-educated caregivers (19–21).

Of note, despite the generally positive attitude toward vaccination, the major reason that could depart from this was the issue of vaccine adverse events mostly observed in infants within 3 months of birth (22). These adverse events according to the caregivers were mostly observed in Oral Polio vaccine/Pentavalent/Rotavirus (given at 6, 10, and 14 weeks of life) followed by OPV/BCG (given at birth). The major source of information regarding vaccination adverse reactions was from health workers during antenatal and child welfare clinics. This is also the major source that contributes to the caregiver’s knowledge regarding vaccination and has the potential to positively improve vaccination uptake (23). One important way for Ghana to improve the strides in vaccination uptake is to make good use of these cost-effective early morning health talks through standardized vaccination information. This vaccination information is not standardized between regions (24, 25). Standardized vaccination information dissemination during early morning health talks in clinics can improve vaccination uptake.

Despite this, more than half of the study population had completed the EPI program for children. However, the main reason for incomplete vaccination in the remaining was time constraints (26, 27). In contrast to other studies, this was commonly observed in highly educated women compared to those with secondary and less formal education (28). It could be explained that the demands of corporate jobs could have been attributed to this. However, it can be curtailed if most official jobs can allocate some free time for caregivers eligible for the EPI program to adhere to their vaccination schedules.

Another major issue observed was that most of the caregivers were unaware of the immunization calendar. Some could not determine when such information was readily available in their child immunization record books (recorded in weeks after delivery). It will be worthwhile if health workers provide a calendar in addition to what is already available or write the next vaccination due date eligibly for caregivers, and provide further education to them on the use of other vaccination information in the record books. This will help curtail the fears of adverse events, and proper time record on the vaccinations they need to complete, when, how, and what to expect.

It was observed that most of the education received by caregivers on vaccination was from health workers, media, family, and friends. The media being a source of information on immunization for caregivers should be cautioned. Importantly, it must be ensured that the right information or standardized vaccination information is being disseminated. This was also positive since it implies that healthcare professionals can collaborate with media agencies to raise awareness on vaccination as well as vaccine adverse events to educate the public on the benefits of vaccination for a community, vaccination schedules, and vaccine follow-ups. Importantly, it will ensure that the right information is disseminated and preventive measures associated with diseases such as HIV are adhered to since some of these mothers even considered HIV to be a vaccine-preventable disease.

A major limitation of the study is the small sample size which was mainly due to time limitations since the study was conducted in partial fulfillment of a medical degree at the University of Ghana. However, in conclusion, the study shows that more than 50% of the caregivers had poor knowledge of vaccination. In addition, other factors that promoted vaccine hesitancy included misinformation on vaccine adverse events, time constraints, and poor use of information provided in child immunization record books. Importantly, we observed that neither social status nor education level impacted the completion of immunization in this group of caregivers. This implies that more awareness needs to be created and involving the media in this will be very useful.

## Data availability statement

The original contributions presented in the study are included in the article/supplementary material, further inquiries can be directed to the corresponding author.

## Ethics statement

The studies involving humans were approved by Ethical and Protocol Review Committee, University of Ghana Medical School, Ghana. The studies were conducted in accordance with the local legislation and institutional requirements. The participants provided their written informed consent to participate in this study.

## Author contributions

SD, NS, and MO conceived the idea and designed the experiments. NS supervised the study. AF assisted in the experimental design. SD, AF, and MO wrote the manuscript. All authors contributed to the article and approved the submitted version.

## Conflict of interest

The authors declare that the research was conducted in the absence of any commercial or financial relationships that could be construed as a potential conflict of interest.

## Publisher’s note

All claims expressed in this article are solely those of the authors and do not necessarily represent those of their affiliated organizations, or those of the publisher, the editors and the reviewers. Any product that may be evaluated in this article, or claim that may be made by its manufacturer, is not guaranteed or endorsed by the publisher.
